# Historical-qualitative analysis of breastfeeding trends in three OECD countries

**DOI:** 10.1186/s13006-019-0230-0

**Published:** 2019-08-06

**Authors:** Amanda Marie Lubold

**Affiliations:** Department of Multidisciplinary Studies, Indiana State University, Holmstedt Hall, 290, Terre Haute, IN 47809 USA

**Keywords:** Breastfeeding initiation, Breastfeeding duration, Qualitative analysis, Public health initiatives, Family policies

## Abstract

**Background:**

Breastfeeding rates among high income, western countries vary considerably. This research examines three countries, Sweden, Ireland, and the United States, with respect to both public health initiatives and policy initiatives.

**Methods:**

This article uses a historical qualitative analysis of breastfeeding rates over time. It uses the welfare state structure as a framework for understanding the variation in breastfeeding outcomes among these three countries.

**Results:**

With its strong family policies and early adherence to international public health recommendations, Sweden was able to build high rates of breastfeeding initiation and duration. However, Sweden’s breastfeeding rates have been declining, which may be a result of increasing encouragement for fathers to take equal leave, and because Sweden is no longer prioritizing breastfeeding in its public health goals. Ireland has experienced rapid growth of both breastfeeding initiation and participation in the Baby-Friendly Hospital Initiative, though its rates still lag behind much of the Western world. The United States has seen increases in participation with the Baby-Friendly Hospital Initiative, but lacks state support in public health and labor policies.

**Conclusion:**

This analysis suggests that in a country with a strong welfare state and early adoption of internationally recommended public policy, breastfeeding is able to flourish. It also suggests that the Baby-Friendly hospital Initiative is a predictor of breastfeeding success.

## Background

In the 1970’s, breastfeeding rates in much of the developed world were experiencing a steady decline [1–3]. Aggressive manufacturing and marketing by infant formula companies, combined with cultural shifts, facilitated a move away from breastfeeding and towards formula feeding, leading international public health organizations to address the decline of breastfeeding [1–3]. However, while breastfeeding rates worldwide have increased since the 1970’s, they have done so at varying rates. What led some countries to increase their breastfeeding rates rapidly, while other countries stagnated? This analysis will examine the factors that led to the differential distribution of breastfeeding rates among three countries, Ireland, Sweden, and the United States, following and expanding upon the work of Galtry in 2003 [4]. Galtry examined the effects of labor market policy and sociocultural factors on breastfeeding rates in three high-income, Organisation for Economic Cooperation and Development (OECD) countries, Sweden, the United States, and Ireland. Galtry specifically chose three countries with vastly different breastfeeding rates in 1997: Ireland at 38% breastfeeding initiation, the United States at 64%, and Sweden at 97%. While rates of breastfeeding have increased in all three countries since Galtry’s research, the disparities among the three countries remain [4]. Galtry focuses primarily on parental leave and workplace and childcare policies in the three nations and finds that more generous parental leave, combined with early childhood programs that encourage and facilitate breastfeeding, can be a supportive model for women [4]. This research builds on by adding analysis of Galtry’s conclusions, and adds the additional dimension of a public health component and a focus on international breastfeeding initiatives, specifically the Baby-Friendly Hospital Initiative.

Available data for breastfeeding incidence and duration are historically plagued with problems of collection and comparability. Recognizing this dearth of information, the European Union (EU) in conjunction with the World Health Organization (WHO), released a “Blueprint for Action” for the “Protection, Promotion, and Support of breastfeeding in Europe” in 2004 [5, 6]. The EU member states received questionnaires on breastfeeding rates and promotion in their countries, and the Blueprint serves as an aggregation of this information into a multi-faceted plan for action to increase breastfeeding promotion across Europe. As part of this plan, the public health arm of the European Commission included breastfeeding as one of its 88 indicators of health for the 2008–2013 Health Programme [7].

The three countries included in the analysis are Sweden, Ireland, and the United States. These three countries parallel the work of Galtry [4] as they represent the three major ideal types of welfare regimes as described by Esping-Andersen [8] and they encompass a wide variety of breastfeeding initiation and duration rates. This analysis examines developments over time in the countries, allowing for consideration of potential indicators that cannot be captured in quantitative indicators. Breastfeeding is a biocultural behavior, and public health initiatives that address biological prerequisites for successful establishment, such as encouraging skin-to-skin in the hospital or maternity center, may make labor market policy redundant if not addressed in early days.

**Fig. 1:**
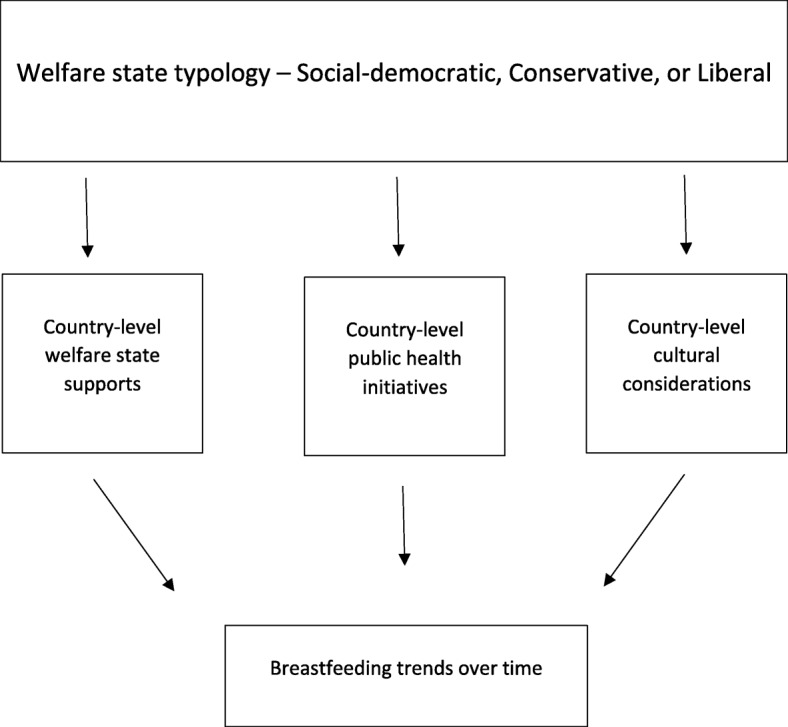
Conceptual model

This research examines two primary veins of policy, welfare state policies and public health initiatives, that often overlap where breastfeeding is concerned. For example, the Innocenti Declaration (referred to hereafter as the Declaration) ratified in 1990, contains provisions that speak to both welfare state policies, including legislation supporting breastfeeding mothers in the workplace, and public health polices, including the Baby-Friendly Hospital Initiative (BFHI) [3, 9]. The Declaration involves four main components, three of which relate directly to public health initiatives, while one addresses national level family welfare policies. This analysis focuses first on degree of compliance with the Declaration, enumerating the relationship between family policies and public health initiatives specifically targeting breastfeeding. The analysis also considers changes over time in maternity leave policy, the Baby-Friendly Hospital Initiative, and national level breastfeeding committees and support. Each of these trends are nested within the analysis of the welfare state typology, while considering their broader impacts between typologies. See Fig. 1 for a conceptual model of the analysis.

In their comprehensive 2016 systematic review of breastfeeding determinants, Rollins et al. identified three main categories of analysis for providing an “enabling environment” for breastfeeding: structural, settings, and individual [10]. The structural determinants include broad level sociocultural and market factors that can shape breastfeeding attitudes and intentions at the macro level. Settings can include both healthcare settings and systems as well as the workplace environment. Individual factors, then, include the mother-infant dyad and their unique attributes [10]. This research paper looks primarily at the structural and settings determinants in the analysis of breastfeeding outcomes across Ireland, Sweden, and the United States.

The basic research question this paper seeks to investigate is “what led some countries to increase breastfeeding initiation and duration, while others stagnated?” This paper identifies two potential hypotheses: 1. The welfare regime is the primary indicator of a country’s value of and commitment to women’s unpaid work, which provides the necessary structure to support breastfeeding or 2. Public health initiatives are more important that labor market policies because breastfeeding is a biocultural activity that must be established early, hence BFHI and other interventions affect successful breastfeeding establishment.

### Trends in breastfeeding outcomes

**Fig. 2:**
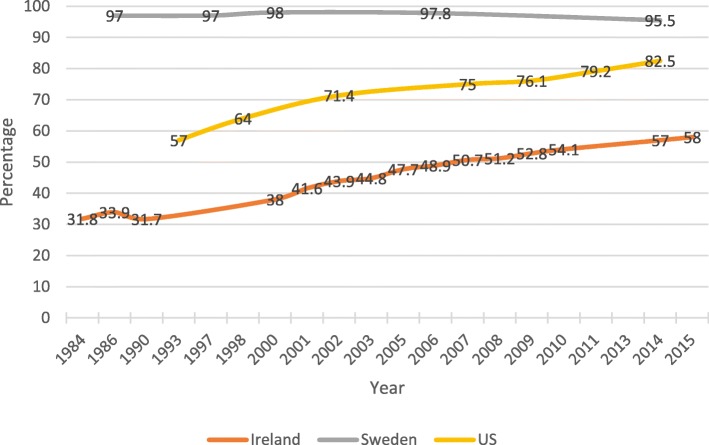
Proportion of women who initiated breastfeeding in Ireland, Sweden, and the US from 1984 to 2015. Sources: [4, 11–19]

Breastfeeding outcomes, both initiation and duration, have been increasing steadily among high-income countries since the late 1970’s [1, 3]. However, in some countries, breastfeeding rates have increased at a much faster rate than in others. The three countries in this analysis have widely differing patterns of changes in breastfeeding rates. Figure 2 displays breastfeeding initiation data over time for the three countries in the analysis [4, 11–19]:

Tables 1, 2, 3 display breastfeeding duration for each country.

**Table 1:** Sweden breastfeeding duration, percentages

Year	4 months any	6 months any
1972		5
1980		50
1986	67.9	50.7
1990	70.2	52.6
1994	79.8	67.3
1998	83.8	73
2000	83.2	72.2
2003	79.9	72.4
2004	82.7	71.9
2006	80.9	69.2
2009		65
2014	74.2	63

[13, 14, 19]

**Table 2:** Ireland breastfeeding duration, percentages

Year	3 months exclusive	3 months any
1995	12	
2004	12.7	
2014		35.4%

[17, 20]

**Table 3:** United States breastfeeding duration, percentages

Year	3 months exclusive	6 months any
1998		29
2000		34.2
2001		36.9
2002		36.9
2003	29.6	39.1
2004	31.5	42.1
2005	32.1	
2006	33.6	43.5
2011	40.7	49.4
2013	44.4	51.8

[16, 21]

Some countries only consider exclusive breastfeeding in reports of breastfeeding duration, while others report mothers who are breastfeeding at all, regardless of whether solid foods or formula are also given. It is difficult to directly compare breastfeeding rates because of the vast differences in data collection, but this analysis will discuss changes over time and compare breastfeeding duration rates when possible.

## Methods

This analysis employs a historical qualitative analysis of major public policy decisions and welfare state climate in Sweden, Ireland, and the United States. It begins with a discussion of welfare state regimes and continues with an analysis of public health and policy initiatives. The paper considers each country individually, analyzing the breastfeeding trends individually, and then looking for commonalities or differences between the three countries. The analysis is limited to social and health system policies. Issues of political economy, specifically dairy production, may also be drivers of breastfeeding trends; however, this research is intentionally narrow as to focus on the specific international public health initiatives. Future research would certainly include a discussion of these economic trends.

### Welfare state regimes

Gosta Esping-Andersen identified three ideal welfare state regimes: social democratic, conservative, and liberal [8]. The social democratic welfare regime, present in most Scandinavian countries, including Sweden, offers a system in which many social protections are extended to working-class and middle-class families. The state also provides a variety of family supports to all citizens. Benefits are neither dependent on the market nor tied to the social class of the recipient [8].

Conservative, or corporatist welfare states, including Ireland, provide some social supports and financial benefits to mothers, but fewer universal benefits to all citizens. Also, the level of support for family work in the form of subsidized or public day care is very limited. Countries in the conservative regime offer long leaves that could encourage motherhood, but then effectively keep mothers out of the labor market because of their low level of support for child care.

The United States is part of the liberal welfare regime. In the liberal regime, the state provides very few social supports and financial benefits to families, instead leaving those supports and benefits up to the markets [8]. The United States is a prime example of a more market-based approach to social welfare. The few state benefits that are available are typically means tested and limited to individuals with great need. Thus, the liberal regime takes a laissez-faire approach to supporting reproductive labor.

In studying breastfeeding through a welfare state model, however, it is important to consider the gendered implications of Esping-Andersen’s ideal types and the more recent research by Orloff and others on gendering analyses of the welfare state. Orloff examines the welfare state model with respect to both gendered labor and care work [20]. Breastfeeding is a uniquely gendered type of care, one that can serve as an ideal example for the necessity of a gendered analysis of welfare states. Women’s economic opportunities are hindered in several ways when they engage in caring activities. First, if women take time out of the paid labor force to perform care work, they can suffer an earnings penalty, often seen in a lifetime earnings gap [20]. Second, mothers in particular face significant challenges in combining motherhood and attachment to the paid labor force [21]. Boeckmann, et al. find that individual or household level differences are not sufficient to explain cross national differences in mothers’ working hours and attachment to the paid labor force. They find that specific policy measures, paid, job protected leave and generous benefits are associated with greater attachment to the labor force and greater working hours for mothers [21].

### Public health initiatives

#### The Innocenti declaration

The Innocenti Declaration on the Protection, Promotion, and Support of Breastfeeding, ratified by the World Heath Organization (WHO) and the United Nations Children’s Fund (UNICEF) in 1990 remains the standard upon which subsequent breastfeeding policies are based. The Innocenti Declaration stated that all countries should adhere to the following four policies to increase breastfeeding:

1. All participating member-state governments should develop national breastfeeding policies and appoint a national breastfeeding coordinator
2. All participating member-state governments should ensure that maternity hospitals and facilities follow the Ten Steps to Healthy Breastfeeding
3. All participating member-state governments should adopt the principles of the International Code of Marketing of Breast-milk Substitutes
4. All participating member-state governments should enact legislation to protect the breastfeeding rights of working women [3].

Each of the three countries included in this study has had varying degrees of compliance with the Innocenti Declaration. Sweden was an “early adopter” of these international recommendations, which is an important component of that country’s successful pathway to high breastfeeding rates.

*Innocenti Declaration Part 1 National Breastfeeding Policies/National Breastfeeding Coordinator.*

The World Health Organization and UNICEF recommended that countries should implement a national breastfeeding policy that includes at least four basic components:

1. Policies should encourage mothers to start breastfeeding soon after birth
2. Policies should encourage mothers to breastfeed exclusively for six months
3. Policies should encourage mothers to breastfeed up to 2 years of age and beyond
4. Policies should implement the Ten Steps for Successful Breastfeeding [3].

Table 4 displays each country with the year a national breastfeeding committee was implemented, the components included in the policy, and whether there is a national breastfeeding coordinator.

**Table 4:** Adherence to part 1 of innocenti declaration

	Year National Breastfeeding Committee Established	Policy Encourages mothers to breastfeeding soon after birth?	Policy encourages mothers to breastfeed exclusively for 6 months?	Policy encourages mothers to breastfeed up to 2 years of age?	Policy implements the Ten Steps for Healthy Breastfeeding?	National Breastfeeding Coordinator?
Ireland	1994	No	Yes	No	Yes	Yes; 2001
Sweden	Voluntary group; 1973 National Committee; 2009	Yes	Yes	No	Yes	No
United States	1998	Yes	No	No	Yes	Yes; 1998

[6, 22, 23]

*Innocenti Declaration Part 2 Ensure that maternity hospitals and facilities follow the Ten Steps to Healthy Breastfeeding.*

The Baby-Friendly Hospital Initiative certifies hospitals with maternity facilities based on their adherence to the Ten Steps to Healthy Breastfeeding.

Tables 5 and 6 show the percentage of hospitals in each country that have been designated as Baby-Friendly over the past fifteen years and the percentage of births in BFHI facilities.

**Table 5:** Percentage of hospitals designated as Baby-Friendly

Country	1997–1998	2000–2001	2003–2004	2006–2007	2009–2010	2016
Ireland	0	0	0	30	35	47
Sweden	91	97	97	97	97	0
United States	0	1	1	3	4	12

Note: Sweden no longer participates in the BFHI program [24–26]

**Table 6:** Percentage of births occurring in BFHI-certified hospitals

	2007	2017
Ireland		47%
Sweden		
United States	2.9%	25.5%

Note: Data were unavailable for Ireland in 2007 and for Sweden [27, 28]

*Part 3 Adopt the principles of the International Code of Marketing of Breast-milk Substitutes.*

Every country except the United States that attended the 1983 World Health Assembly ratified the International Code of Marketing of Breast-milk Substitutes, but few countries actually enacted the provisions into law. Sweden is the only country in this analysis that adopted many provisions of the code into law [29].

*Part 4 Enact Legislation to Protect the Breastfeeding Rights of Working Women.*

The last part of the Innocenti Declaration declares that countries should protect the breastfeeding rights of working women [3]. The International Labor Organization, in their 2000 Maternity Protection Convention, the most up-to-date standard, recommended that countries should guarantee paid maternity leave of at least 14 weeks, ensure that women get nursing breaks, and limit restrictions and exclusions of women from these laws [30].

#### Summary

This analysis will examine the pathways to breastfeeding “success” within each country, following the conceptual model, Fig. 1, which examines country level welfare state policies, public health initiatives, and cultural considerations couched in broader welfare-state regimes. Broader welfare state regimes can inform all three of these country level variables. For example, countries in the social democratic welfare regime provide more generous supports, but also support greater regulation of product markets, labor markets, and the health system.

## Results

### Social-democratic welfare regime: Sweden

As Fig. 2 and Table 1 illustrate, the Scandinavian countries that represent the Social-Democratic welfare regime like Sweden have very high rates of breastfeeding initiation and duration. Breastfeeding initiation rates in Sweden have remained consistently high over the past decade. In 1997, breastfeeding initiation was at 97%, and in 2007 initiation was at 97.8% [1, 15]. Sweden also has high rates of female labor force participation (61% in 2017) [31]. Sweden was an early adopter of many facets of the Innocenti Declaration, which created a strong public health initiative to encourage breastfeeding. Sweden also provides substantial maternity leave support that subsidizes income and provides job protected time out of the workplace, to encourage mothers to establish breastfeeding [32, 33].

### Public health initiatives

The Swedish International Development Authority (SIDA), a Swedish government-sponsored organization, was one of the co-sponsors of the WHO/UNICEF conference in Florence, Italy, called “Breastfeeding in the 1990s: A Global Initiative,” where the Innocenti Declaration was adopted [34]. As one of the early supporters of the WHO/UNICEF initiatives, Sweden has adopted many of the recommendations and provisions regarding breastfeeding. The Swedish Nursing Mother’s Support group (Amningshjalpen) is a non-profit, voluntary group of women to support nursing mothers. The group was created in 1973, and its goals are to support breastfeeding women, and to provide information and a breastfeeding culture in Sweden [35]. The Swedish Nursing Mother’s Support group has been influential in helping to implement breastfeeding policy, including the Baby-Friendly Hospital Initiative. This voluntary group was the only breastfeeding committee until 2009, when Sweden established a National Breastfeeding Committee, attached to the National Board of Food Administration [24].

With 97% of its hospitals and maternity facilities designated as Baby-Friendly since 2000, Sweden has consistently adhered to the joint directive from WHO/UNICEF. Sweden was among the first countries to adopt the Baby-Friendly Hospital Initiative. In 1997, WHO and UNICEF introduced the Baby-Friendly Hospital Initiative, and by 1997, 91% of hospitals in Sweden were designated Baby-Friendly [13, 25]. By 2000, 97% of hospitals in Sweden were Baby-Friendly. However, in 2004, the national authority responsible for administering the BFHI, the Swedish National Institute of Public Health, stopped overseeing the initiative, and individual regional coordinators were supposed to continue administration. Elisabeth Kylberg, a member of the National Swedish Breastfeeding Committee, comments that this plan did not come to fruition, and administration of the BFHI stalled nationwide [24].

Sweden adopted the International Code of Marketing Substitutes in 1983, making it one of the first two countries (along with Norway) to do so [36]. Many provisions of the Code are law in Sweden, and in 2013, Sweden’s parliament voted to restrict further advertising and marketing of infant formula only to scientific publications and specialized baby care publications [33]. In addition, the 2013 law restricts formula companies from providing free or low cost formula to mothers, except in cases where it is medically necessary [37]. These provisions were in place after the adoption of the Code in 1983, but only on a voluntary basis. By adopting the provisions into law, Sweden further strengthened its commitment to the WHO/UNICEF recommendations.

It is also relevant to consider the role of cultural shifts within Sweden and how those shifts affect breastfeeding norms, expectations, and then overall breastfeeding trends. The percentage of foreign-born individuals in Sweden has increased from 11.3% in 2000 to 18.5% in 2017 [38]. As the demographics of the country shift, cultural norms and breastfeeding expectations across the population may become less homogenous and thus more variable.

### Welfare state supports

Swedish parents have 50 weeks of parental leave, paid at 80% of their regular income [39]. The parental leave is flexible, it can be divided between both parents, and can also be taken in half day or one-quarter day increments. The leave benefits have no requirement for job tenure or to have paid into the system, but parents who have been contributing to the insurance system receive a higher level of benefits. There is also a “gender equality bonus,” instituted in 2008, that provides an additional cash benefit to families if parents share parental leave [32, 39]. Mothers, specifically, are guaranteed 14 weeks of dedicated maternity leave [40].

Sweden’s breastfeeding rates are among the highest in the world. In 1997, 97% of mothers had initiated breastfeeding, and in 1998, 73% of mothers were continuing to breastfeed at six months [4]. This was an increase from a 91.6% initiation rate and a 50.7% breastfeeding rate at six months in [13]. However, since 1997, breastfeeding initiation rates have held steady, while breastfeeding duration has been decreasing. In 1998, 74.8% of infants were breastfed at six months of age; by 2009 that number had dropped to 64.8% [14]. Likewise, in 1997, 92.6% of infants were breastfeeding at two months of age, the highest incidence, but by 2009 only 88.1% of infants at two months old were being breastfed [14].

### Summary and additional considerations

One factor that may account for decrease in breastfeeding duration is Sweden’s shift of focus in the public health arena away from breastfeeding. In 2003, Sweden adopted a public health policy which focused on eleven domains, breastfeeding is not mentioned in Sweden’s newest public health policy documents [41]. Sweden also dropped national control of the Baby-Friendly Hospital Initiative in 2004; in 1992, the BFHI was administered by the National Board of Health and Welfare in Sweden, and then from 1997 to 2003, the Swedish National Institute of Public Health administered the initiative [24]. However, in 2004, there was no longer a national authority that agreed to administer the program, and the BFHI was dropped. Hospitals in Sweden are still certified as Baby-Friendly, but there is no active national organization continuing to administer it. In 2009, a National Breastfeeding Committee was developed in Sweden, and they are actively petitioning various governmental agencies to restart the initiative [24]. Sweden’s failure to continue to administer the BFHI at a national level appears to have an effect on overall breastfeeding rates, especially duration. Sweden has a welfare state model that supports a dual-earner structure, where women are fully integrated into the labor force, but also are given sufficient maternity leave and state supports to support care work. However, even given the supportive environment for women in Sweden, breastfeeding rates are on a slight decline, and have not been able to keep up the robust numbers of the late 1990s and early 2000s. It is thus relevant to question whether this failure to maintain high rates despite a favorable welfare state model means that the welfare state model may not be the driving factor in enabling high breastfeeding rates.

As an added consideration, Sweden has been increasingly encouraging fathers to take a larger share of the paid parental leave. In 1995, Sweden introduced a month of paid leave reserved only for fathers, called “daddy’s month” [40]. In 2002, Sweden extended paternal leave to two months, paid at 80% [40]. In order to encourage mothers and fathers to split leave equally, Sweden also has introduced a “Gender Equality Bonus.” This program offers an incentive for fathers to take more of the available leave allotted to each parent by providing each parent a financial incentive for each day they use leave equally [40]. As such, the downward trends in breastfeeding duration may be a reflection of the recent push for fathers to take more leave. If fathers are taking more of the shared leave, mothers may be returning to work earlier, which could lead to a decline in breastfeeding duration.

## Conservative welfare regime: Ireland

Ireland has the lowest breastfeeding initiation rate among high-income OECD countries. In Ireland 31.4% of mothers initiated breastfeeding in 1984, and by 2015, initiation increased, but still only 58.0% of Irish mothers initiated breastfeeding [11, 17]. Ireland also has relatively low rates of female labor force participation, 53%, so women are less attached to the labor force than in countries in other welfare regimes [31]. Policies in Ireland tend to support women’s reproductive labor in isolation from productive labor, while not encouraging attachment to the labor market. The burden of all care work, then, is squarely on the shoulders of mothers, who are not engaged in the traditional labor market, instead gaining their rights and positions in society through the informal care sector. Why, then, are breastfeeding rates so low? One potential explanation is that Ireland falls into the “one and a half breadwinner” welfare typology. Women are responsible for the care, but they also are increasingly in the paid labor force, albeit as part-time workers. Indeed, the female share of part-time work in Ireland in 2017 was 72.2%, which is among the highest rates in OECD countries [42]. Because of the lack of state support for care, women are finding themselves in a double bind, they are still responsible for the traditional caring responsibilities at home, but are also working part-time in the labor force with limited political or financial power.

### Public health initiatives

Ireland has provided some public health supports for breastfeeding, but it has only been in the last decade that these policies have been implemented in any meaningful way. In 1991, Ireland implemented a voluntary agreement based on the International Code of Marketing of Breast-Milk Substitutes. The agreement is limited in nature and only covers basics of labelling and advertising [43]. Advertising of formula is restricted in the voluntary agreement, but the scope is limited and enforcement is spotty. The Food Safety Authority of Ireland (FSAI) is responsible for monitoring the manufacturers and organizations, but little enforcement has been done. In fact, a report in 2003 stated that 34% of new mothers surveyed had received commercial gift packs from hospitals, and 81% had their names and addresses recorded by formula manufacturers [43].

The first National Breastfeeding Policy for Ireland was published in 1994. It provided recommendations and targets for improving breastfeeding rates. The 1994 policy followed the recommendations of WHO and UNICEF, including the International Code on the Marketing of Breastfeeding Substitutes, the Innocenti Declaration, and the Baby Friendly hospital Initiative [44].

Ireland adopted the Baby-Friendly Hospital Initiative in 1998. They appointed a national breastfeeding coordinator in 2001 and established the National Committee on Breastfeeding in 2002. Volunteer groups such as La Leche League and Cuidiu-Irish Childbirth Trust made an impact on breastfeeding rates, according to the Interim Report of the National Committee on Breastfeeding [44].

Ireland’s breastfeeding rates have been steadily increasing since 2001, but overall rates remain low. However, the rise of breastfeeding rates seems to parallel the rise of hospitals in Ireland designated as Baby-Friendly. In 2001, Ireland’s breastfeeding initiation rate was 41.6%, and by 2015 the initiation rate was 58.0% [12, 17].

In 2005, Ireland’s National Committee on Breastfeeding developed an action plan for increasing breastfeeding. Ireland’s public health goals for breastfeeding follow the guidelines of the Ottawa Charter from the World Health Organization in 1986. The development of health promotion practices and policy at international, national, and local levels is guided by the Charter. It defines promoting health as “the process of enabling people to increase control over, and improve their health” [23].

The goals of Ireland’s Breastfeeding Action Plan are [45]:

- All families have the knowledge, skills, and support to make and carry out informed infant feeding decisions, particularly those least likely to breastfeed
- The health sector takes responsibility for developing and implementing evidence based breastfeeding policies and best practice
- Communities support and promote breastfeeding in order to make it the normal and preferred choice for families in Ireland
- Legislation and public policies promote, support, and protect breastfeeding
- Irish society recognizes and facilitates breastfeeding as the optimal method of feeding infants and young children.

The plan also included targets [45]:

- Target 1: Data collection – the development of a comprehensive, accurate and timely infant feeding data collection
- Target 2: Breastfeeding rates – increase initiation by 2% per year and 4% per year for lower SES groups. Increase duration by 2% per year and 4% per year for lower SES groups. Will be measured at 3 months, 6 months, and 12 months
- Target 3: Baby Friendly Hospital Initiative – at least 50% of hospital births will take place in Baby friendly hospitals and 100% of hospitals will be Baby Friendly by five years from the start date.
- Target 4: Regional breastfeeding coordinators – implement 10 coordinators by October 2006

### Welfare state supports

Mothers in Ireland are guaranteed 42 weeks of maternity leave total, with the first 26 weeks paid at 80% of the recipient’s pretax wages [39]. The remaining 16 weeks is unpaid, but still job-protected. While there is no job tenure requirement for the maternity leave provision, mothers do have to have contributed to the insurance fund for at least 39 of the previous 52 weeks before taking it. There is no paid paternity leave, but mothers and fathers both have access to 14 weeks of unpaid leave, and they can take it any time up to the child’s eighth birthday [32, 33]. Between 90 and 100% of women in Ireland are covered under the maternity leave law [40].

Ireland is part of the conservative welfare regime, which was shaped partly by the traditional male-breadwinner model. Ireland has the lowest breastfeeding rates, both initiation and duration, of any country in this study, and in fact, of any high income, OECD country. Despite fairly generous maternal leave entitlements and a public health commitment to breastfeeding that dates back to at least 1990, Ireland has struggled to raise its rates of breastfeeding. Ireland has increased its breastfeeding initiation rates since they have begun implementing the Baby-Friendly Hospital Initiative. As of 2017, 9 of Ireland’s 19 hospitals and maternity centers are certified Baby-Friendly, up from 0% as recently as 2004 [25, 46]. While breastfeeding rates have been increasing, they are still the lowest among high-income, OECD countries. Ireland has a large female share of part time employment (72.2% in 2017), and so despite maternity leave entitlements that support women’s reproductive labor and generous family benefits, women still are not fully attached to the traditional labor market, and are working part-time in addition to their caregiving responsibilities [42].

### Summary and additional considerations

In September 2017, the National Committee of the Baby Friendly Health Initiatives suddenly announced that it would be ending its activities in Ireland [47]. This was a sudden development in response to news that the HSE (Health Service Executive), Ireland’s health services had dropped the grant services it was providing to the BFHI in Ireland [47]. Despite the fact that Ireland has seen sustained growth in both percentage of babies born in BFHI facilities and overall breastfeeding rates, the funding was dropped. It will be important to follow this development, as the breastfeeding rates in Ireland continue to lag behind much of the west, but they have increased at a sustained rate over the last decade and a half. Considering that despite welfare state typology, initiation and duration of breastfeeding have been increasing (albeit at different rates) among high income OECD countries, the availability and continued increase and maintenance of BFHI facilities may show to be a more robust finding. As described earlier, Sweden’s well established high rates of breastfeeding appear to be on a slight decline, which coincides with BFHI no longer being overseen at the national level. While this trend is not causative, it is potentially indicative of a trend to watch.

In addition, the role of culture must be considered, even among an analysis of welfare state supports. Pfau-Effinger (2005) and Aboim (2010) examine the ways in which cultural understandings of gender shape and inform welfare state typology [48, 49]. Indeed, Aboim notes, “[w]ithout culture, it would be difficult do grasp why … there are such different developments in women’s participation in the labour force and, furthermore, why the organization of family life has responded so differently to women’s entry into the paid labour force” (p. 177) [49]. In couching breastfeeding within a welfare state context, the role of culture and gendered expectations also must be considered. In Ireland, cultural beliefs around breastfeeding may contribute to Ireland’s lower breastfeeding rates. For example, Tarrant et al. (2009) found significant differences in breastfeeding initiation between Irish-born mothers and non-Irish nationals [50]. Irish born mothers in the Dublin sample had a 47% initiation rate, compared to non-Irish nationals living in Dublin, whose initiation rate was 79.6% [50]. These significant differences point to the role of cultural expectations around breastfeeding for Irish mothers, which may be more robust than a structural, welfare-state regime effect. Indeed, Tarrant and Kearney (2008) note that because of the lack of a breastfeeding culture in Ireland, public health initiatives to support mothers once they leave the maternity hospital, not just in the hospital, must be robust if the breastfeeding rates are to see a significant increase [51].

## Liberal welfare regimes – United States

The United States, adhering to the ideal type of the liberal, or market-based welfare regime, tends to provide support for women’s attachment to the labor force, but has lower levels of support for women’s reproductive labor. For example, the United States provides no nationally mandated paid maternity leave, which makes it difficult for women to provide care for their infants and also continue to be economically active [32, 33, 52].

### Public health initiatives

The United States has had a tenuous history with the WHO/UNICEF breastfeeding recommendations. The United States endorsed the Code in 1994, but no provisions of the Code are law, and marketing of formula is unregulated. In fact, the United States was the sole “no” vote when the International Code was passed, 118 to 1, in 1981 [53]. Infant formula has a long history of marketing in the United States; until the late 1980s, formula was marketed to healthcare professionals, who then supported and recommended certain formula brands to women in healthcare facilities [54]. However, starting in the late 1980s, formula companies began marketing directly to women, through magazines, commercials, and other direct-to-consumer means. As Shealy et al. pointed out, the United States has a longstanding tradition of free speech and advertising, and that is at odds with the recommendations of the Code [54]. The United States Food and Drug Administration (FDA) regulates infant formula nutrient content, and companies that wish to market formula in the United States must register with the FDA. However, the FDA is not responsible for overseeing or regulating any marketing efforts [43]. The United States’ limited adoption of the Code and unwillingness to regulate infant formula marketing is also in line with the market-based, or liberal welfare state. As part of the liberal welfare state, the United States tends to rely on the market to regulate social policies.

The United States Breastfeeding Committee (USBC) was founded as a tax-exempt nonprofit in 2002, seven years after the planning started [22]. The committee was formed in response to the Innocenti Declaration, but it took 12 years from the Declaration until the USBC was founded. The USBC was planned and drawn up by a group of volunteers committed to increasing breastfeeding. The USBC has been active in setting up breastfeeding coalitions in all 50 U.S. states. However, these coalitions are often voluntary and do not have state or federal funding [22]. In 2000, the United States Centers for Disease Control (CDC) launched *Healthy People 2010*, a national initiative targeting a wide variety of health-related indicators. This was a follow up to *Healthy People 2000*, a similar program launched in 1990 [36]. *Healthy People 2010* marked the first time that breastfeeding was included as an objective under the target public health objective of improving maternal, infant, and child health [36]. The objectives include increasing any breastfeeding, breastfeeding at six months, breastfeeding at one year, and exclusive breastfeeding at both three and six months [36].

### Welfare state supports

The United States has no paid maternity leave. Until 1993, there was no nationally mandated, job protected leave for new mothers in the United States. In 1993, the Family and Medical Leave Act (FMLA) was passed, which provides for 12 weeks of unpaid, job protected leave for new mothers. The 12 weeks of leave can also be taken due to illness, illness of a family member, or by fathers after the birth of a child. The FMLA is unique among high income countries, first because the leave is unpaid, and second because the leave is not exclusively for the birth of a child and can also be used for illness related leave. In order to be eligible for FMLA leave, the recipients must have worked for at least one year in their job, and the job must be with an employer that has at least 50 workers [32, 33]. Indeed, the ILO reports that only about 33–65% of women are covered under any federal maternity protections [40].

The United States, as part of the liberal welfare regime, has a laissez-faire approach to many of the WHO/UNICEF recommendations, preferring instead to promote self-regulation and market-regulation of many of these breastfeeding-supportive policies. The United States has seen an increase in breastfeeding initiation and duration since the 1970s, but its rates still lag behind WHO recommendations [15]. The Family and Medical Leave Act of 1993 marked the first true governmental policy designed to address maternity leave, but this legislation came much later than legislation supporting maternity leave in other similarly-situated countries, and is not specific to maternity. The United States was late to adopt the Baby-Friendly Hospital Initiative, and as of 2010, only 4% of hospitals with maternity wards were designated Baby-Friendly [25]. This lack of adherence to the BFHI ties in with the United States’ welfare state typology, because formula companies have free reign to advertise and provide samples to mothers, it is increasingly difficult to certify hospitals as Baby-Friendly. The goals and objectives of major WHO/UNICEF recommendations such as the BFHI and the Code are at odds with a welfare state that promotes market-based solutions to family issues. In the United States, the maternity leave entitlement structure supports women’s productive labor, but does not support their reproductive labor. Women have no paid maternity leave, and job protected, unpaid leave was only introduced in 1993. As part of the liberal welfare regime, the United States, as general policy, entrusts the markets to take care of family related leave and supports.

### Summary and additional considerations

In 2018, the United States caused a small international incident when it initially refused to ratify a resolution at the World Health Assembly in Switzerland [55]. The resolution stated that mother’s milk was the healthiest for children, and that countries should continue to protect and promote breastfeeding [55]. This action seems to follow the United States’ well documented lack of support for international level public health initiatives surrounding breastfeeding. However, the strides the United States is making in certifying Baby-Friendly hospitals could shed some positive light on their interventions to increase breastfeeding.

## Conclusion

In this analysis trends in breastfeeding rates over time were compared among three countries: the United States, part of the liberal welfare regime; Ireland of the conservative welfare regime; and Sweden, part of the social democratic regime. In the early 1970’s, breastfeeding rates in all of these high-income countries were very low, but by the mid 2000’s, breastfeeding outcomes varied widely throughout the three countries. The differential policy regimes have led to the differential development of breastfeeding outcomes. In Sweden, breastfeeding increased rapidly because of policies supporting women’s productive and reproductive labor, valuing care work as a public good, and supporting international public health initiatives as public support for care. In Ireland, care is still considered the domain of women, and there is less in the way of public support for care. This lack of recognition of care work as a valued public good has resulted in lagging adherence to international public health initiatives targeting breastfeeding. Combined with the constraints women face in Ireland due to the “one and a half breadwinner” model, breastfeeding rates have remained stagnant.

The results of this analysis suggest several theoretical implications in both gendered welfare state theory and public health literature. First, Sweden as an early adopter of international recommendations has the highest breastfeeding rates. Compliance with the WHO/UNICEF initiatives, however, depends on welfare regime policies and overall support for women in both productive and reproductive labor. For example, Sweden was an early adopter of the Baby-Friendly Hospital Initiative and many parts of the Innocenti Declaration. Sweden also has policies that recognize women as both contributors to the labor market and as valued providers of care work. In social democratic welfare countries, women gain their rights and positions in society through *both* productive and reproductive labor, and care work is supported as a valued public good.

Second, results suggest that the Baby-Friendly Hospital Initiative may have an effect on breastfeeding rates. For example, Sweden, which has 97% of its hospitals certified as Baby-Friendly has not kept up with institutionalized control of its Baby-Friendly Hospital Initiative since 2004, and many of its hospitals are failing to get recertified. Since 2004, the duration of breastfeeding in Sweden has also decreased from a high of 72.4% of women breastfeeding at six months in 2003 to only 65% in 2009. Ireland, on the other hand, increased its Baby-Friendly Hospital Initiative participation throughout the 2000’s, and Ireland’s breastfeeding initiation has increased from 38% in 2000 to 54.1% in 2010.

This study does have several important limitations. First, understanding and teasing out mechanisms of change is extremely complex in an environment where multiple interlocking factors are at play. The findings of this study cannot be considered causative; instead, the point of this research is to examine how and why the welfare state and public health initiatives work together (or not) in providing an enabling environment for breastfeeding. This study also does not consider the role of social movements in change. The BFHI in particular may be considered a type of social movement, and that literature is not addressed in the research. Finally, the role of non-governmental organizations (NGOs) and other grassroots organizations is not explored. Examining the influence and capacity of breastfeeding NGOs is a third factor that may be relevant in each country’s specific pathway.

Future research on this topic would address the question of breastfeeding as a social movement, specifically looking at the roles of NGOs and other stakeholders. In addition, it would be useful to perform in-depth interviews with key figures in each country, such as the breastfeeding coordinator or members of the breastfeeding committee. In addition, it would be useful to examine other similarly situated countries in each of the three main welfare state regimes to examine the role of public health factors within a similar broader welfare structure.

## Data Availability

Data used in this study are available upon reasonable request. Please contact the author for data requests.

## Abbreviations

BFHI: Baby-Friendly Hospital Initiative
CDC: Centers for Disease Control
EU: European Union
FDA: Food and Drug Administration
FMLA: Family and Medical Leave Act
HSE: Health Service Executive
NGO: Non-governmental organizations (NGOs)
OECD: Organisation for Economic Co-operation and Development
SIDA: Swedish International Development Authority
UNICEF: United Nations Children’s Fund
USBC: United States Breastfeeding Committee
WHO: World Health Organization
